# Editorial: The gut microbiome and COVID-19

**DOI:** 10.3389/fcimb.2023.1213346

**Published:** 2023-05-24

**Authors:** Tao Lin, Jennifer Lin, Amy Sims

**Affiliations:** ^1^ Department of Molecular Virology and Microbiology, Baylor College of Medicine, Houston, TX, United States; ^2^ Baylor College of Medicine, Houston, TX, United States; ^3^ Pacific Northwest National Laboratory, Richland, WA, United States

**Keywords:** COVID-19, microbiome, viral load, disease severity, dysfunctional immune responses

## The microbiome and human health/disease

Over the past two decades, microbiome studies confirmed the importance of microbiota to human health and diseases and ongoing studies are focused on the mechanism(s) of how the microbiota impacts and responds to disease and the immune response. To date, only a small percentage of the bacteria in the human microbiota have been isolated, identified, and studied because the required growth conditions could not be reproduced in the laboratory. However, recent technological advances have made it feasible to analyze the entire human microbiome and study host-microbiota interactions. The application of state-of-the-art technologies, such as *in vivo* mono-colonization, functional assays in germ-free (GF) and genetically modified mouse models, *in vitro* co-cultures of the anaerobic microbiota and aerobic host tissues, spatial resolution, and ‘meta-omics’-related research (such as metagenomics, transcriptomics, dual-transcriptomics, metabolomics, and proteomics) have provided major insights into the complexity of relationships between the host and microbiota as well as the functional roles of the microbiota and individual bacteria in host health and disease. These techniques have been used to study the mechanisms of host microbiota interactions and facilitated the discovery and development of microbiome-related therapeutics.

## The gut microbiota is associated with viral load, disease severity, and dysfunctional immune responses

The outbreak of the severe acute respiratory syndrome coronavirus 2 (SARS-CoV-2) and its rapid international spread with ongoing devastating effects resulted in a global health crisis. SARS-CoV-2 is likely to continue as a pandemic threat into the future. SARS-CoV-2, the causative agent of the coronavirus disease-2019 (COVID-19) pandemic, was first identified in China and is thought to have emerged from reservoir host bats into presumed intermediate hosts and then transmitted into humans.

Since COVID-19 broke out, multiple variants of SARS-CoV-2 have been isolated and identified in different countries and are circulating globally. Examples include Alpha (B.1.1.7, initially detected in the United Kingdom and around 50% more transmissible than the original Wuhan strain), Beta (B.1.351, first detected in South Africa, around 50% more transmissible than previous variants, and is associated with more severe disease), Gamma (P.1, initially identified in Brazil and 1.7-2.4 times more transmissible than non-variants), B.1.427 and B.1.429 (first identified in California), Delta (B.1.617.2, consists of two mutations on the spike protein of the virus, responsible for the rapid increase of infections in India, and 40-60% more transmissible than the Alpha variant), and Omicron (B.1.1.529, first detected in South Africa, has a large number of mutations, causes an increased risk of reinfection, and could be partially resistant to existing COVID-19 vaccines). These emerging variants have played a role in the increased global spread of COVID-19 (O’Toole et al., 2021).

SARS-CoV-2 virus enters host cells through angiotensin-converting enzyme 2 (ACE2) receptors. The virus mainly infects lung epithelial cells, but epithelial cells in the gastrointestinal tract, kidneys, cardiovascular system, and neuro system are also permissive for infection. Evidence indicates that the gut microbiota can alter SARS-CoV-2 virus load and COVID-19 severity (Zuo et al., 2020) and that ACE2 regulates the gut microbiota (Viana et al., 2020). Recent studies show that intestinal infection with SARS-CoV-2 correlates with the dysbiosis of the gut microbiota as well as the severity of COVID-19 symptoms (Mancabelli et al., 2022). Moreover, some gut microbiota may enhance host antiviral immunity and stimulate interferon production. Thus, the gut microbiota could be an effective tool for diagnosing and treating COVID-19.

Gastrointestinal symptoms are present in many COVID-19 patients. COVID-19 patients showed a significantly reduced richness and diversity of gut microbiota, a significantly higher abundance of opportunistic pathogens (e.g., *Candia albicans*) and short-chain fatty acid-producing bacteria, a highly heterogeneous mycobiome configuration, lower abundance of beneficial symbionts, immune deregulation, and delayed SARS- CoV-2 clearance. The correlation between the altered microbiota from fecal samples and the elevated levels of intestinal inflammatory cytokine IL-18 was observed in the serum of COVID-19 patients. The gut microbiota alteration might also contribute to SARS-CoV-2-induced cytokine storms.

## Successful collection of microbiome and COVID-19 studies

The goal of this Research Topic is to provide a platform for articles that aim to grow our knowledge base on the relationship between COVID-19 and the human gut microbiome. Some fascinating studies were published on this Research Topic, including 5 original research articles, one brief research report, one perspective, and one hypothesis. These articles found important associations between the microbiome and COVID-19, including the gut fungal microbiome in COVID-19 patients (Reinold et al., 2022), detection of SARS-CoV-2 in COVID-19 patients’ feces (Wu et al., 2022), bile inhibits SARS-CoV-2 endoribonuclease Nsp15 activity in the mouse gut (Ma et al., 2022), the relationship between pediatric gut microbiota and SARS-CoV-2 infection (Romani et al., 2022), low production of endogenous hydrogen gas in COVID-19 patients (Ostojic, 2022), COVID-19 severity associations with population-level gut microbiome variations (Lymberopoulos et al., 2022), the contribution of the gut-brain axis to the development of neurological symptoms in COVID-19 recovered patients (Vakili et al., 2022), and alterations in the intestinal DNA virome in COVID-19 patients (Lu et al., 2021).

These research articles explored the relationship between the microbiome and COVID-19 at different levels, from epidemiological studies to viral detection and the association of the microbiome with viral load, disease severity, and immune dysregulation. For example, one study (Reinold et al., 2022) found both immune dysregulation and gut fungal microbiota dysbiosis to be associated with severe/critical SARS-CoV-2 infections. Reduced gut fungal diversity and richness as well as increased abundance of a single fungal species in the *Ascomycota* phylum were observed in severe COVID-19 cases.

Another interesting study explored fecal-oral transmission of COVID-19. Viral shedding in feces has not been completely investigated. This original research article explored the COVID-19 virus in patients’ feces and found that 35.1% of patients showed detectable SARS-CoV-2 RNA in their feces (Wu et al., 2022). The median time of viral shedding in feces was approximately 25 to 33 days.

The gut microbiome profile of COVID-19 patients has also found to be correlated with viral load, disease severity, and dysfunctional immune responses. To investigate the role of gut microbiota in limiting/preventing SARS-CoV-2 infection, Ma et al. established a high-throughput *in vitro* screening system and found that bile inhibits SARS-CoV-2 endoribonuclease Nsp15 activity in the mouse gut.

Romani et. al. focused on how SARS-CoV-2 infection affects children’s gut microbiota. The article reported that children infected with COVID-19 showed gut microbiota dysbiosis, with lower α-diversity and a specific β-diversity. *Faecalibacterium* and reduced fatty acid and amino acid degradation were also observed in pediatric COVID-19 patients, possibly associated with reduced severity of SARS-CoV-2 infected children.

Other recent studies also suggest that the human gut microbiome might be associated with COVID-19 severity. Lymberopoulos et al. explored the association between the human gut microbiome and the severity of COVID-19 by analyzing hospitalization rates from different countries. They found that COVID-19 severity is associated with population-level gut microbiome variations. Twelve countries were divided into two groups (high/low) according to COVID-19 hospitalization rates. The authors reported an association of distinct gut microbiome signatures with COVID-19 severity. The results suggest an association of anti-inflammatory bacteria, such as *Bifidobacteria* species and *Eubacterium rectale*, with lower severity, while pro-inflammatory bacteria, such as *Prevotella copri*, are associated with higher severity.

COVID-19 infection might also change the homeostasis of the gaseous bioactive molecule dihydrogen (H2) produced by the human gut bacteria. The molecular footprints of COVID-19 could be tracked in the family of biologically active gases and gas transmitters. Studies have shown various alterations of the gut microbiota in COVID-19 patients, including a lower abundance of hydrogen-producing bacteria. Dihydrogen has many important biological functions, such as cytoprotective, antioxidant, anti-inflammatory, and antiapoptotic roles. A perspective article (Ostojic, 2022) suggests that the low production of dihydrogen in COVID-19 patients might contribute to disease progression and severity and that exogenous administration of dihydrogen could be beneficial for COVID-19 patients. H2 supplementation to restore normal levels could thus aid COVID-19 diagnosis and treatment.

A hypothesis article reviewed the contribution of the gut-brain axis to the development of neurological symptoms in COVID-19 patients (Vakili et al., 2022). The authors suggest that the long-term neurological symptoms of COVID-19 may be related to intestinal microbiota disorders and describe several mechanisms through which this gut dysbiosis can lead to long-term neurological disorders. These mechanisms may be mediated by cytokines as well as certain chemicals such as gastrointestinal hormones (e.g., CCK), neurotransmitters (e.g., 5-HT), bacterial products (e.g., short-chain fatty acids), and the autonomic nervous system.

## Conclusion

The emergence of SARS-CoV-2 and its rapid international spread pose a global health disaster. Research on the mechanisms of how the gut microbiome impacts SARS-CoV-2 viral load, COVID-19 pathogenesis, and COVID-19 severity is a fledgling field and will be a flourishing research area. The knowledge and techniques gained from these studies will benefit COVID-19 control, prevention, diagnosis, and treatment. We have collected several attractive papers on this Research Topic and hope to see more exciting studies and emerging techniques soon.

## Author contributions

All authors listed have made a substantial, direct, and intellectual contribution to the work and approved it for publication.

## References

[B1] Mancabelli L. Milani C. Fontana F. Lugli G. A. Tarracchini C. Viappiani A.. (2022). Untangling the link between the human gut microbiota composition and the severity of the symptoms of the COVID-19 infection. Environ. Microbiol. 24 (12), 6453–6462. doi: 10.1111/1462-2920.16201 36086955 PMC9538590

[B2] O’Toole Á. Scher E. Underwood A. Jackson B. Hill V. McCrone J. T.. (2021). Assignment of epidemiological lineages in an emerging pandemic using the pangolin tool. Virus Evol. 7 (2), 1–9. doi: 10.1093/VE/VEAB064 PMC8344591 34527285

[B3] Viana S. D. Nunes S. Reis F. (2020). ACE2 imbalance as a key player for the poor outcomes in COVID-19 patients with age-related comorbidities – role of gut microbiota dysbiosis. Ageing Res. Rev. 62, 1–13. doi: 10.1016/J.ARR.2020.101123 PMC7365123 32683039

[B4] Zuo T. Zhang F. Lui G. C. Y. Yeoh Y. K. Li A. Y. L. Zhan H.. (2020). Alterations in gut microbiota of patients with COVID-19 during time of hospitalization. Gastroenterology 159 (3), 944–955.e8. doi: 10.1053/j.gastro.2020.05.048 32442562 PMC7237927

